# The Impact of Metabolic Syndrome on Immune Regulation (IL-17, IL-23, and FOXP3+), Psoriasis Severity, Flare Frequency, and Quality of Life in Psoriasis Patients: A Cross-Sectional Study

**DOI:** 10.1155/ijin/5855171

**Published:** 2025-06-20

**Authors:** Flora Ramona Sigit Prakoeswa, Faradiba Maharani, Saiful Hidayat, Winda Atika Sari, Triasari Oktavriana, Cita Rosita Sigit Prakoeswa, Menul Ayu Umborowati, Ratih Pramuningtyas, Rochmadina Suci Bestari, Riandini Aisyah, Erika Diana Risanti, Listiana Masyita Dewi, Ilham Hafizha Maulana Anam

**Affiliations:** ^1^Department of Dermatology Venerology and Aesthetic, Faculty of Medicine, Universitas Muhammadiyah Surakarta, Surakarta, Central Java, Indonesia; ^2^PKU Muhammadiyah Hospital, Surakarta, Indonesia; ^3^Faculty of Medicine, Universitas Sebelas Maret, Surakarta, Central Java, Indonesia; ^4^National Heart and Lung Institute, Imperial College London, London, UK; ^5^Department of Cardiovascular Medicine, Graduate School of Medicine, Kobe University, Kobe, Japan; ^6^Department of Dermatology Venerology and Aesthetic, Universitas Sebelas Maret Hospital, Surakarta, Central Java, Indonesia; ^7^Department of Dermatology Venerology and Aesthetic, Faculty of Medicine, Airlangga University, Surabaya, East Java, Indonesia; ^8^Department of Dermatology Venerology and Aesthetic, Dr. Soetomo General Academic Hospital, Surabaya, Indonesia

**Keywords:** FOXP3+, IL-17, IL-23, immune dysregulation, metabolic syndrome, psoriasis

## Abstract

**Introduction:** Psoriasis is a chronic inflammatory skin disease that exhibits a strong association with metabolic syndrome (MetS). The involvement of various proinflammatory cytokines in MetS is thought to play a critical role in the pathogenesis of psoriasis. This study aims to evaluate the impact of MetS on immunological markers (IL-17, IL-23, and FOXP3+ regulatory T cells), disease severity, and quality of life (QoL) among patients with psoriasis.

**Methods:** This cross-sectional study involved 42 psoriasis patients, divided into two groups: 29 without MetS (Pso) and 13 with MetS (Pso-MetS). Clinical parameters such as blood pressure, fasting blood glucose, and triglyceride levels were measured. Immunological markers (IL-17, IL-23, and FOXP3+) were analyzed using ELISA. Psoriasis severity was assessed using the Psoriasis Area and Severity Index (PASI), and QoL was evaluated with the Dermatology Life Quality Index (DLQI).

**Results:** The Pso-MetS group was significantly older than the Pso group (*p* value = 0.003). Higher systolic (*p* value < 0.001), fasting glucose (*p* value = 0.002), and triglycerides (*p* value < 0.001) were observed in the Pso-MetS group. Lower HDL observed in the Pso-MetS group (*p* value = 0.004). FOXP3+ expression was significantly lower in the Pso-MetS group (*p*=0.02), while waist circumference, diastolic blood pressure, IL-17, IL-23, PASI, and DLQI scores levels showed no significant differences.

**Conclusions:** MetS is associated with immune dysregulation, evidenced by reduced FOXP3+ expression in psoriasis patients. Further studies are needed to explore the immunological link between psoriasis and MetS.

## 1. Introduction

Psoriasis is a chronic autoimmune disease with primary cutaneous manifestations, characterized by hyperstimulation of keratinocytes, leading to visible lesions on the skin [1]. Globally, psoriasis affects approximately 2%-3% of the population, with significant regional variation. In European countries, the prevalence can reach 8%–11%, whereas in Asian populations, it is generally lower, ranging between 0.2% and 1.5% [2–4]. In Indonesia, the exact national prevalence is still underreported, but hospital-based studies suggest a growing trend in dermatology outpatient visits due to psoriasis [5, 6].

Over the past decade, increasing evidence has highlighted a strong link between psoriasis and metabolic syndrome (MetS)—a cluster of conditions including central obesity, hypertension, dyslipidemia, and hyperglycemia. A population-based study in the United Kingdom involving 6549 psoriasis patients showed a significantly higher prevalence of MetS (34%) compared to matched controls (30%), with the risk increasing alongside psoriasis severity [7, 8]. Similarly, a study in India by Nisa and Qazi (2010) involving 100 age- and sex-matched controls found a MetS prevalence of 28% among psoriasis patients versus 6% in controls, supporting this association in South Asian populations [9]. These findings suggest that systemic inflammation inherent in psoriasis may exacerbate the metabolic dysregulation observed in MetS [10, 11]. Moreover, the chronic inflammatory state induced by MetS may worsen the severity and frequency of psoriasis flares, potentially creating a vicious cycle that negatively impacts patient outcomes [11].

Although psoriasis primarily affects the skin, its impact on patients' quality of life (QoL) extends far beyond physical symptoms, influencing emotional well-being, social interactions, and daily functioning [12]. Patients with psoriasis, particularly those with comorbid conditions such as MetS (Pso-MetS), often experience significantly diminished QoL due to the compounded burden of managing both skin lesions and systemic complications such as cardiovascular disease and diabetes [13]. The co-occurrence of psoriasis and MetS has shown to exacerbate the physical and emotional burden of disease. Patients with both conditions may experience increased fatigue, joint pain, body image disturbances, and a higher risk of depression and anxiety, all of which contribute to a greater impairment in QoL compared to those with psoriasis alone [14]. This dual impact makes understanding and addressing QoL in psoriasis patients, especially those with MetS, critical for developing holistic treatment approaches that target both the physical and emotional aspects of these conditions.

Despite the well-established association between psoriasis and MetS, the specific immunological mechanisms linking these two conditions remain underexplored. It is hypothesized that the IL-23/Th17 axis, a key player in the pathogenesis of psoriasis, also plays a significant role in the development and progression of MetS [10]. Moreover, regulatory T-cells (Tregs), identified by FOXP3+ expression, might be dysregulated in patients with both conditions, further contributing to the chronic inflammation observed [10, 15]. Dysregulation in these pathways not only drives disease progression but also provides a rationale for targeted therapies aimed at restoring immune balance through inhibition of IL-23/Th17 signaling or enhancing Treg function.

This study aims to investigate the impact of MetS on immune regulation, psoriasis severity, flare frequency, and QoL in patients with psoriasis. Focusing on the expression levels of key cytokines such as IL-17, IL-23, and FOXP3+, this research seeks to uncover potential immunological mechanisms that could inform future personalized treatment strategies.

## 2. Methods

### 2.1. Study Design

This study was conducted as an observational analytic study with a cross-sectional approach. Data collection took place from March to August 2024 at the Dermatology and Venereology Clinic of Universitas Sebelas Maret Hospital, involving patients diagnosed with psoriasis who met the predetermined inclusion and exclusion criteria.

### 2.2. Study Population and Sample Size

This study initially targeted a sample size of 50 psoriasis patients, divided into two groups: those with and without MetS. However, due to challenges in data collection, we obtained complete data from 42 psoriasis patients, with 13 patients in the Pso-MetS group (psoriasis with MetS) and 29 patients in the Pso group (psoriasis without MetS). The selection of study subjects adhered to the inclusion and exclusion criteria. Inclusion criteria were patients aged ≥ 18 years who were currently receiving standard psoriasis therapy, including topical corticosteroids, systemic treatments such as methotrexate (MTX), or phototherapy (narrow-band ultraviolet B [NB-UVB] or laser). Exclusion criteria included patients with other autoimmune comorbidities, terminal illnesses, or those currently hospitalized.

### 2.3. Diagnosis of MetS

MetS was diagnosed if three or more of the following criteria according to the guidelines developed by the 2001 NCEP ATP III and American Heart Association (2005) [16] met:

• Central obesity: Waist circumference > 102 cm in men or > 88 cm in women.
• Hypertension: Blood pressure > 130/85 mmHg.
• Fasting blood glucose (FBG) ≥ 100 mg/dL.
• Plasma triglycerides ≥ 150 mg/dL.
• Low HDL cholesterol: < 40 mg/dL in men or < 50 mg/dL in women.

### 2.4. Blood Draw Collections

Venous blood samples were collected after an 8-h fast by a trained phlebotomist using standard aseptic techniques. Blood was drawn from the antecubital vein with sterile needles and vacuum tubes:

• Serum Separator Tubes (SST): For serum analysis of fasting glucose, triglycerides, and HDL cholesterol using automated enzymatic assays (glucose oxidase, glycerol-3-phosphate oxidase, and colorimetric methods).
• EDTA Tubes: For plasma collection to measure immunological markers (IL-17, IL-23, and FOXP3+) via ELISA, following the manufacturer's instructions. FOXP3+ levels were assessed from plasma samples, not from whole blood. The ELISA kits used were obtained from Bioassay Technology Laboratory (Shanghai, China), with detection ranges of 7.8–500 pg/mL for IL-17, 31.25–2000 pg/mL for IL-23, and 0.156–10 ng/mL for FOXP3+. Sensitivity and specificity were based on the validation data provided by the manufacturer.

### 2.5. Clinical Assessment of Psoriasis

The severity of psoriasis was assessed by board-certified dermatologist using the Psoriasis Area and Severity Index (PASI), a standardized tool for measuring the extent and severity of psoriatic lesions. The frequency of psoriasis flares will be recorded based on patient history over the past year. The impact of psoriasis on the patients' QoL was assessed using the Dermatology Life Quality Index (DLQI).

### 2.6. Data Analysis

Normality of data distribution was assessed using the Shapiro–Wilk test. Categorical variables were expressed as frequencies and percentages. Continuous variables were presented as mean ± standard deviation or median (interquartile range), depending on data distribution. Group comparisons were conducted using independent *t*-tests or Mann–Whitney *U* tests. Therapeutic groups (topical, oral, and phototherapy) were compared with PASI scores to evaluate treatment response. All statistical analyses were performed using STATA Version BE 18.

## 3. Results

This study enrolled 42 psoriasis patients, comprising 29 individuals without MetS (Pso) and 13 individuals with both psoriasis and MetS (Pso-MetS). The analysis revealed significant differences in key clinical, biochemical, and immunological parameters between the two groups.

### 3.1. Clinical Parameters

Patients in the Pso-MetS group were significantly older than those in the Pso group (56.5 ± 11.5 vs. 41.3 ± 12.6 years, *p*=0.003). Systolic blood pressure was also significantly higher in the Pso-MetS group (154.3 ± 18.97 mmHg vs. 124.9 ± 17.73 mmHg, *p* < 0.001), whereas diastolic pressure showed no significant difference (91.2 ± 12.3 vs. 84.48 ± 10.33 mmHg, *p*=0.273).

### 3.2. Biochemical Parameters

The Pso-MetS group had significantly higher FBG and triglyceride levels compared to the Pso group. The mean FBG was 137.08 ± 64.97 mg/dL vs. 82.03 ± 9.44 mg/dL (*p*=0.002), and triglyceride levels were 263.92 ± 173.67 mg/dL vs. 110.89 ± 32.3 mg/dL (*p* < 0.001). Conversely, HDL levels were significantly lower in the Pso-MetS group (46.46 ± 6.91 mg/dL) than in the Pso group (53.1 ± 32.3 mg/dL, *p*=0.004), as shown in Table 1.

### 3.3. Immunological Parameters

A significant difference was observed in FOXP3+ expression, which was lower in the Pso-MetS group (0.027 ± 0.020) compared to the Pso group (0.064 ± 0.067, *p*=0.022). This suggests impaired Treg function in patients with MetS, which may contribute to persistent inflammation. In contrast, no significant differences were found in IL-17 (3.38 ± 7.26 vs. 5.57 ± 19.67 pg/mL, *p*=0.663) or IL-23 (10.11 ± 4.80 vs. 8.92 ± 7.49 pg/mL, *p*=0.157). The lack of significance may be attributed to the small sample size, variability in immune response, and potential confounding from ongoing treatments, which can modulate cytokine levels, as seen in Table 1.

### 3.4. Psoriasis Severity and QoL

No significant differences were found between the groups in PASI or DLQI scores. The mean PASI score was 11.76 ± 12.26 in the Pso group and 10.34 ± 9.31 in the Pso-MetS group (*p* value = 0.99). Similarly, DLQI scores did not differ significantly (*p* value = 0.095).

### 3.5. Treatment Patterns and PASI Scores

As seen in Table 2, the most common therapy in the Pso group was phototherapy combined with MTX and cream (*n* = 8, PASI = 13.76), followed by MTX with cream and biologic agents (*n* = 6, PASI = 8.1), and MTX with folic acid and desoximetasone cream (*n* = 6, PASI = 5.23). Phototherapy with MTX alone (*n* = 5) was associated with the highest PASI score (26.9), likely reflecting more severe cases. One patient received no treatment, with a PASI of 0.3.

In the Pso-MetS group, phototherapy with MTX was also the most common regimen (*n* = 7, PASI = 13.03). Two patients received only topical therapies such as desoximetasone or vaseline (PASI = 3.55), and three patients were untreated (PASI = 7.26). No patients in this group received biologic agents or MTX–cream combinations.

These findings suggest more limited use of systemic or biologic therapies in the Pso-MetS group, potentially due to comorbidity-related considerations. Overall, PASI scores tended to be lower in the Pso-MetS group across most treatment types. However, formal statistical analysis between therapy groups was not conducted due to the small and uneven distribution of patients across treatment subgroups, which limits the power to detect meaningful differences. Therefore, the data are presented descriptively to illustrate general trends and generate hypotheses for future research.

## 4. Discussion

This study showed that psoriasis patients with MetS (Pso-MetS) are significantly older than those without MetS (Pso), corroborating previous studies that reported a similar results [17]. Advanced age in Pso-MetS patients may amplify chronic systemic inflammation, which in turn contributes to both metabolic dysregulation and skin pathology. In addition, longer disease duration and immune system aging (immunosenescence) could influence immune marker expression and treatment response [9, 18, 19]. Central obesity characterized by an increased waist circumference is a primary component of MetS in psoriasis patients [20]. A study by Ferdinando et al. found that a significantly larger waist circumference is more common in psoriasis patients with MetS [17]. The lack of significant findings regarding waist circumference in this study could be attributed to the relatively small sample size or variations in fat distribution within the studied population.

In terms of flare frequency, disease severity and QoL were assessed using PASI score and DLQI. This study found that no significant differences were found in PASI and DLQI scores between the psoriasis patients with and without MetS. A previous study involving 20 Pso-MetS patients and 22 Pso patients showed similar PASI score results to this study, where higher PASI scores were observed in the group without MetS, although the results were not statistically significant [21]. These nonsignificant results could reflect a disconnection between systemic metabolic status and localized skin inflammation. Studies show that while psoriasis can affect mental health and social relationships, patients with MetS may focus more on cardiovascular and metabolic complications rather than skin-related issues [22]. As a result, PASI scores and reductions in QoL as reflected in the DLQI may not be significantly impacted by skin disease alone. Furthermore, a study by Barrea et al. noted that the DLQI scoring system allows patients to mark certain questions as “not relevant,” which are then scored as zero [13]. This could lead to lower DLQI scores that do not necessarily reflect the true severity of the disease, as lower scores may stem from patients finding certain daily activities less relevant rather than experiencing minimal impact from their condition.

Systolic blood pressure demonstrated a significant difference in this study although diastolic blood pressure did not show statistical significance. These results align with the findings of Botelho et al. who observed that hypertension is more prevalent among psoriasis patients with MetS compared to controls [23]. Systemic inflammation marked by elevated levels of CRP, TNF-α, and IL-6 has been linked to increased vascular stiffness and endothelial dysfunction, which tend to elevate systolic pressure more than diastolic [24, 25]. This not only exacerbates psoriasis-related inflammation but also contributes to endothelial dysfunction and insulin resistance [24]. Moreover, patients with Pso-MetS exhibited significantly higher FBG and triglyceride levels, consistent with established associations between psoriasis and insulin resistance in MetS. Elevated triglyceride levels, in particular, reflect the presence of dyslipidemia—a hallmark of MetS—while reduced HDL levels further increase the risk of vascular inflammation and atherosclerosis [26–28].

IL-17 and IL-23 are both key cytokines in the IL-23/Th17 pathway that drives psoriatic inflammation and metabolic dysregulation [22]. While IL-17 plays a direct role in promoting keratinocyte proliferation and systemic inflammation, IL-23 sustains the Th17 cell population responsible for producing IL-17, IL-22, and TNF-α [22, 29]. In this study, IL-17 levels were lower and IL-23 levels were slightly higher in the Pso-MetS group, but the differences were not statistically significant. This may reflect cytokine variability across individuals, and importantly, the suppressive effects of ongoing systemic or topical treatments on serum cytokine levels.

However, these differences were not statistically significant. Other than unequal distribution of patients between groups and the relatively small sample, circulating IL-17 and IL-23 levels may not necessarily reflect the severity of inflammation in psoriasis patients with MetS. This statement was supported by a meta-analysis by Liu et al. that found no significant correlation between IL-23 levels and psoriasis progression, and Nakajima et al. reported undetectable IL-17 and IL-23 levels in psoriasis and control groups, suggesting that these cytokines might be involved primarily in the early stages of psoriasis or may only show elevated levels in skin lesions [30, 31]. Although lower IL-17 levels were observed in the Pso-MetS group in this study, this finding aligns with the work of Surendar et al. who also observed lower IL-17 levels in patients with MetS compared to controls [32]. This can be explained by the dual effect of TGF-β on IL-17 synthesis: low concentrations of TGF-β stimulate IL-17 secretion, while high concentrations induce inhibition on this activity [32]. Previous research suggests that IL-23 may be more involved in metabolic processes, including glucose homeostasis, rather than in skin inflammation [33]. The nonsignificant IL-17 and IL-23 results in this study indicate that their roles in mediating inflammation in psoriasis and MetS may be more complex. Other studies suggest that IL-17 may have a more dominant role in linking psoriasis with MetS, particularly in its systemic effects, while IL-23 primarily supports Th17 cell activity without directly driving psoriasis or metabolic dysfunction [22].

FOXP3+ is a transcription factor for Tregs that plays a role in maintaining immune system homeostasis and controlling inflammation [34]. Previous studies have suggested a potential role of FOXP3+ in the pathogenesis of psoriasis through the IL-6 and TGF-β pathways [35]. The findings from this research indicate that MetS is associated with reduced FOXP3+ expression, which can lead to impaired immune regulation and heightened inflammation. This aligns with the earlier research that demonstrated decreased FOXP3+ expression in the peripheral circulation of psoriasis patients, although the relationship with disease severity has been inconsistent [36, 37]. In psoriasis patients, FOXP3+ Tregs may undergo differentiation into IL-17A-producing cells, indicating a loss of their regulatory function [35]. This functional shift is critical as it contributes to systemic inflammation and metabolic disturbances, with Th17 cells known to influence metabolic pathways and contribute to conditions such as insulin resistance [11]. The significantly lower FOXP3+ expression observed in the Pso-MetS group in this study suggests a profound impairment in immune regulation, potentially affecting both systemic and local inflammatory control.

The dysregulation of FOXP3+ in Pso-MetS patients suggests that it may serve not only as a biomarker of immune imbalance but also as a potential therapeutic target. Modulating Treg activity or enhancing FOXP3+ expression could offer a dual benefit in controlling both cutaneous and metabolic inflammation. Further exploration of this pathway could open new avenues for immune-modulating treatments in psoriasis with comorbid MetS.

The descriptive analysis presented in Table 2 further supports the clinical relevance of immune-metabolic interactions in psoriasis. Patients without MetS (Pso group) showed more diverse treatment patterns, including combined use of phototherapy, MTX, creams, and even biologics. In contrast, those in the Pso-MetS group predominantly received phototherapy and fewer systemic treatments. This difference may be attributed to clinicians' hesitancy to prescribe aggressive immunomodulatory therapies due to comorbidity risks. Interestingly, the highest PASI scores in the Pso group were observed in those receiving phototherapy and MTX alone, which may indicate that this regimen is typically reserved for more severe or refractory cases. Meanwhile, untreated patients in the Pso-MetS group still demonstrated moderate PASI scores, reflecting a potential undertreatment or clinical decision to withhold therapy due to systemic risk factors.

It is also important to consider that most patients in this study were undergoing some form of treatment, which may have influenced serum cytokine levels. MTX, phototherapy, and topical corticosteroids are known to exert immunosuppressive effects that can modulate IL-17 and IL-23 expression, potentially masking significant differences between groups. Similarly, immunomodulatory therapy may affect FOXP3+ expression by influencing Treg function. Therefore, the observed lack of significant differences in IL-17 and IL-23 levels, as well as the reduction in FOXP3+ expression, must be interpreted with caution, considering the immunosuppressive impact of ongoing treatments. Future studies should consider stratifying patients by treatment status or implementing washout periods to better isolate true immunological differences.

It is also important to consider that most patients in this study were undergoing some form of treatment, which may have influenced serum cytokine levels. MTX, phototherapy, and topical corticosteroids are known to exert immunosuppressive effects that can modulate IL-17 and IL-23 expression, potentially masking significant differences between groups [38]. Similarly, immunomodulatory therapy may affect FOXP3+ expression by influencing Treg function [39]. Given the small sample size and uneven distribution across treatment subgroups, formal statistical analysis was not conducted. Instead, this descriptive approach was adopted to explore treatment patterns and generate hypotheses for future research. Nevertheless, these observations underscore the need for personalized and integrated management strategies that account for both dermatologic and metabolic factors.

## 5. Conclusions

This study demonstrated immune dysregulation in psoriasis patients with MetS, as evidenced by a significant reduction in FOXP3+ expression. The absence of significant differences in IL-17 and IL-23 levels suggests that circulating cytokine levels may not adequately reflect the severity of systemic or cutaneous inflammation, particularly in patients receiving treatment. Due to the small and uneven sample sizes between groups, the results—especially regarding treatment responses—were analyzed descriptively. Future studies with larger, balanced cohorts and longitudinal designs are warranted to better understand the immunometabolic connections in psoriasis and to identify potential therapeutic targets.

## Figures and Tables

**Table 1:** Clinical, biochemical, immunological, and quality of life characteristics of psoriasis patients with and without metabolic syndrome.

Characteristics	Pso (*n* = 29)	Pso-MetS (*n* = 13)	*p* value
Age (years)	41.31 ± 12.62	56.5 ± 11.5	**0.003**
Systolic blood pressure (mmHg)	124.9 ± 17.73	154.3 ± 18.97	**< 0.001**
Diastolic blood pressure (mmHg)	84.48 ± 10.33	91.2 ± 12.3	0.273
Fasting blood glucose (mg/dL)	82.03 ± 9.44	137.08 ± 64.97	**0.002**
Triglycerides (mg/dL)	110.89 ± 32.3	263.92 ± 173.67	**< 0.001**
HDL (mg/dL)	53.1 ± 32.3	46.46 ± 6.91	**0.004**
FOXP3+ (ng/mL)	0.064 ± 0.067	0.027 ± 0.020	**0.022**
IL-17 (pg/mL)	5.57 ± 19.67	3.38 ± 7.26	0.663
IL-23 (pg/mL)	8.92 ± 7.74	10.11 ± 4.80	0.157
PASI	11.76 ± 12.26	10.34 ± 9.31	0.988
DLQI	9.67 ± 6.37	7.23 ± 6.83	0.095

*Note:* Pso = psoriasis without MetS, Pso-MetS = psoriasis with MetS, and FOXP3+ = forkhead box P3. Bold values indicate statistically significant differences (*p* < 0.05) between the groups.

Abbreviations: DLQI = Dermatology Life Quality Index, FBG = fasting blood glucose, HDL = high-density lipoprotein, IL = interleukin, and PASI = Psoriasis Area and Severity Index.

**Table 2:** Types of psoriasis treatment and average PASI scores among patients with (Pso-MetS) and without (Pso) metabolic syndrome.

Main therapy	Pso-MetS (*n*)	PASI score (Pso-MetS)	Pso (*n*)	PASI score (Pso)
Phototherapy + MTX	7	13.03	5	26.90
Phototherapy + MTX + cream	0	0.00	8	13.76
MTX + cream + biologic agent	0	0.00	6	8.10
MTX + folic acid + desoximetasone cream	1	14.40	6	5.23
Others	2	3.55	3	5.40
No therapy	3	7.26	1	0.30

*Note:* MTX = methotrexate, Pso = psoriasis without MetS, and Pso-MetS = psoriasis with MetS.

Abbreviation: PASI = Psoriasis Area and Severity Index.

## Data Availability

The datasets generated or analyzed during the current study are not publicly available but are available from the corresponding author on reasonable request.
